# Shifts in the foraging tactics of crocodiles following invasion by toxic prey

**DOI:** 10.1038/s41598-021-03629-6

**Published:** 2022-01-24

**Authors:** Abhilasha Aiyer, Richard Shine, Ruchira Somaweera, Tina Bell, Georgia Ward-Fear

**Affiliations:** 1 School of Life and Environmental Sciences, University of Sydney, Camperdown, NSW 2006 Australia; 2 School of Biological Sciences, Macquarie University, North Ryde, NSW 2109 Australia; 3 School of Biological Sciences, The University of Western Australia, Crawley, WA 6009 Australia

**Keywords:** Behavioural ecology, Conservation biology, Invasive species, Tropical ecology

## Abstract

Biological invasions can modify the behaviour of vulnerable native species in subtle ways. For example, native predators may learn or evolve to reduce foraging in conditions (habitats, times of day) that expose them to a toxic invasive species. In tropical Australia, freshwater crocodiles (*Crocodylus johnstoni*) are often fatally poisoned when they ingest invasive cane toads (*Rhinella marina*). The risk may be greatest if toads are seized on land, where a predator cannot wash away the toxins before they are absorbed into its bloodstream. Hence, toad invasion might induce crocodiles to forage in aquatic habitats only, foregoing terrestrial hunting. To test this idea, we conducted standardised trials of bait presentation to free-ranging crocodiles in sites with and without invasive toads. As anticipated, crocodiles rapidly learned to avoid consuming toads, and shifted to almost exclusively aquatic foraging.

## Introduction

Invasive species can have catastrophic impacts on native wildlife, and in doing so, can induce changes that increase the ability of the native species to tolerate the presence of the invader (e.g., by developing resistance to toxins, or decreasing preferences for toxic prey^1,2^) Even if population declines are modest, the arrival of an invasive species may induce adaptive shifts in the biology of a native species^3^. For example, a newly arrived predator may impose intense selection against prey individuals that fail to detect or avoid the novel threat^4,5^; or may modify the risk of foraging in specific habitats. The reintroduction of wolves to Yellowstone discouraged elk from feeding in open spaces near rivers (where wolves posed a high risk), transferring grazing preferences to forested areas^6^.

Similar shifts in behaviour might be expected if the vulnerable species is a native predator and the invader is a toxic prey species. The spread of cane toads (*Rhinella marina*) through Australia has led to the fatal poisoning of many predators that ingest this toxic amphibian^7–9^. The toad invasion has not only reduced the abundance of several predator species but has also modified frequency distributions of ecologically significant traits such as sexes, body sizes, and “personality” dimensions such as boldness/shyness within populations of the affected species^10,11^. One puzzling case involves freshwater crocodiles (*Crocodylus johnstoni*), in which some populations have experienced high mortality whereas others have been unaffected by the arrival of cane toads^7,12^. That heterogeneity in impact is not easily explicable in terms of crocodile density, physiological resistance to toad toxins or demography, all of which are broadly similar among populations^13^. Recently, Shine (2018)^14^ recounted a novel hypothesis suggested to him by a citizen scientist, Dave Lindner (see quote at the beginning of this paper). Lindner surmised that the location of encounter between toad and predator affected the outcome. Terrestrial foraging was perilous, because a predator that seizes a cane toad on land cannot wash away the toxin before it is absorbed. In contrast, a predator seizing a toad in the water might be able to flush the toxin away before it enters the predator’s bloodstream. By analogy, the most effective way to save the life of a dog that has seized a cane toad is to flush out the dog’s mouth with copious amounts of water^15^. Thus, aquatic predation might buffer amphibious predators against toad-induced poisoning. This hypothesis fits with other situations where animals (mostly mammals) employ behavioural mechanisms akin to ‘prey washing’, which enable them to consume toxic prey^16^.

We can test this idea in crocodiles by quantifying foraging locations of individuals in sites with and without cane toads (i.e., behind the toad invasion front, *versus* in advance of the invasion front). We predict that terrestrial foraging will decrease in frequency after toads have invaded, for two potential reasons: (a) natural selection, if there is a genetic basis to behaviour or foraging-site choice, and (b) learning, if a crocodile with a non-fatal experience when foraging on land thereafter shifts to predominantly aquatic foraging.

Importantly, the relative frequency of terrestrial *versus* aquatic foraging by a semi-aquatic predator also depends on other factors as well. Thus, to test our prediction we deployed baits in a standardised fashion to remove confounding geographic differences in visibility, abundance, or ease of capture of terrestrial prey compared to aquatic prey. Because the hypothesis relies upon crocodiles adapting to exclude toads from the diet, we also presented a “control” (edible) bait as well as a mildly toxic toad leg, in order to evaluate whether crocodilian prey choice as well as foraging sites were affected by the arrival of cane toads.

## Materials and methods

### Study area

The Kimberley region is in the wet-dry tropics of north-western Australia. This area has strong seasonality in rainfall patterns, usually divided into the ‘wet’ season (November to April) where up to 95% of rainfall occurs (long term wet season average: 798 mm of the total 833 mm annually for Kununurra, Western Australia^17^); and the ‘dry’ season (May to October). Temperatures are high year-round (mean monthly maxima > 30 °C^17^).

Field trials were conducted in two locations: (a) areas invaded by cane toads in 2011 near Kununurra in the east Kimberley (15° 46′ 24″ S, 128° 44′ 21″ E; trials conducted in November 2019), with sites ranging from artificial gravel pits to waterbodies surrounded by dense natural woodland.These were considered the ‘toad-sympatric’ populations; and, (b) areas where cane toads are yet to arrive in the central west Kimberley, Windjana Gorge National Park (17° 24′ 2″ S, 124° 56′ 4″ E; trials conducted in September 2020) with discrete waterbodies located along the Lennard River which runs through sandstone gorges. These were considered the ‘toad naïve’ populations. At each location, we gathered data at five discrete waterbodies (see Supplementary Materials for details). Each waterbody was spotlighted before and during the study to confirm the presence of freshwater crocodiles; waterbodies varied in population densities of crocodiles (toad naïve: n = 4–33 individuals, toad-sympatric: n = 23–46 individuals).

### Study species

The Australian freshwater crocodile (*Crocodylus johnstoni*) is a medium-sized crocodilian; females grow to 2 m snout to vent length (SVL) and up to 40 kg in weight; males to 3 m SVL and up to 100 kg^18,19^. It is the largest freshwater predator in northern Australia^19^. Freshwater crocodiles usually hunt small aquatic and semi-aquatic prey from shallow water, but occasionally and opportunistically also forage on land^18,20^.

### Baiting stations and bait deployment

**Figure 1:**
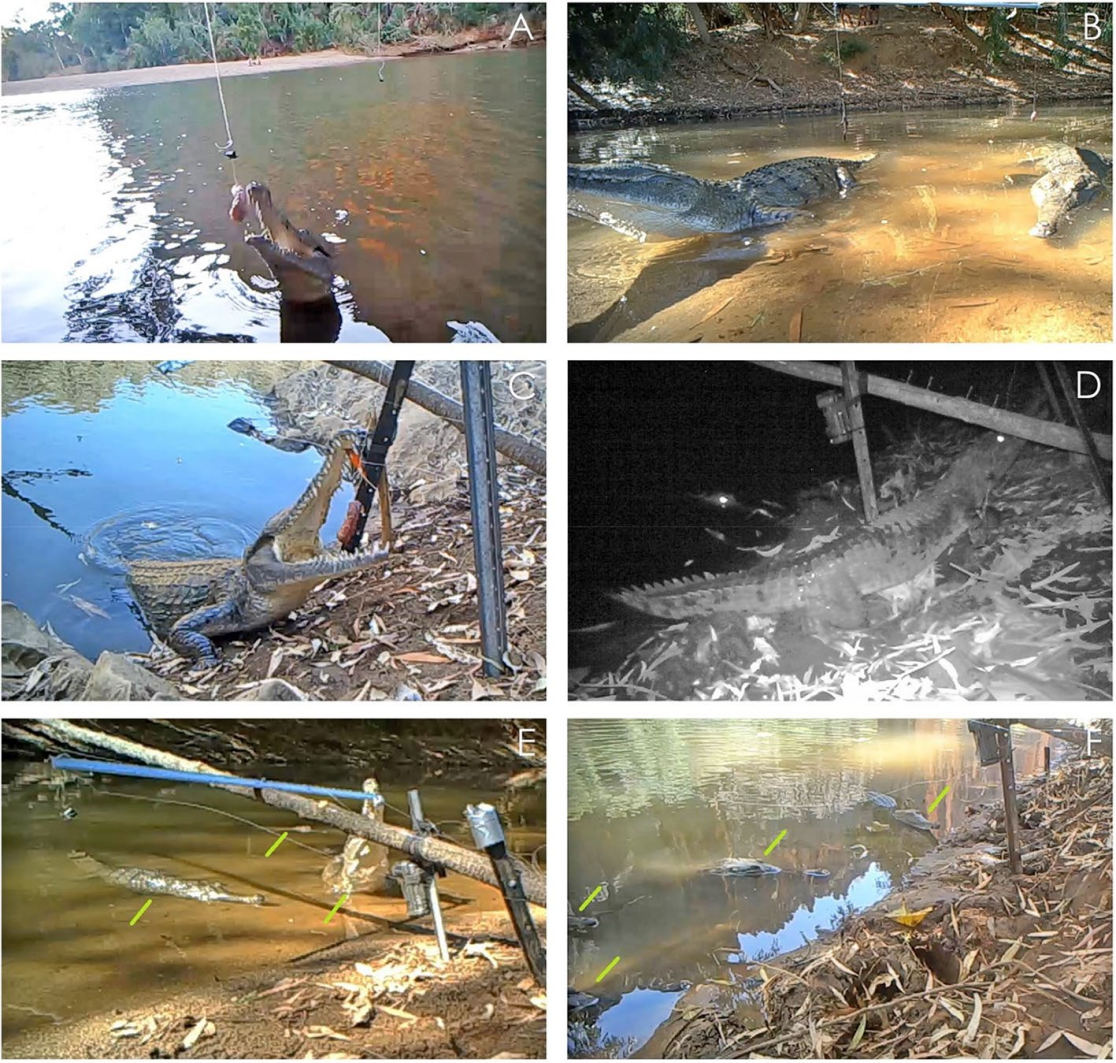
Photographs of freshwater crocodiles taking baits during baiting trials, from the water (**A**,**B**); as well as on land (**C**,**D**), and showing that sometimes multiple crocodiles visited the bait stations concurrently (**E**,**F**; green lines indicate individuals). Photographs taken from remote camera footage and by M. Bruny.

To deploy baits, we used a 3.6 m central wooden pole (~ 50 mm thickness) stretching over the waterbody at an angle from the bank (Fig. 1a; see Supplementary Materials for more details). We suspended baits from cross bars on the central pole at each of two locations: directly over water, and at the edge of the waterbody where the water meets the bank. Baits were also suspended from stakes on the sandy/soil bank in line with the baits on the wooden apparatus to complete the three ‘baiting locations’ available for foraging – forming a complete baiting ‘station’ (Fig. 1; and also see Supplementary Materials for diagram of station set-up). Each location was fitted with a control bait (chicken neck, approximately 50 mm long, 40 g fresh weight) and a treatment bait (rear half of adult cane toad carcass, approximately 50 mm long, 25 g fresh weight). Treatment (toad) baits were non-lethal as internal organs and parotid glands had been removed. Toad baits hence had very little toxin and were unlikely to induce taste aversion^21^. We randomly assigned baits to either side of the station so that visiting animals could choose between both bait types at each location. To suspend the baits, we used natural fibre twine tied around the bait and attached this by ‘bulldog’ clips to the frame, to allow easy release of the bait if pulled from a downward direction (Fig. 1, and see Supplementary Materials). Baits dangled approximately 10 cm above the water or ground surface to allow them to swing freely and minimise consumption by ants or fish (Fig. 1; see Supplementary Materials). Three baiting stations were established at equal distances around each waterbody.

Each bait station was filmed using two remotely triggered camera traps (Model: Ltl Acorn 6310Wmc). One camera trap was attached to the wooden apparatus and directed at baits located over water; the other directed at baits at the edge and on the bank, fixed to a standalone picket behind the station (Fig. 1 and see Supplementary Materials). Cameras were triggered by both motion and infrared thermal detection and were set to take 1-min videos with every trigger.

### Trial period

Baits were deployed at 1700 h on Day 1 and retrieved at 1700 h on Day 5. Video data were collected for eight distinct intervals; four nights (1700–0800 h) and four days (0800–1700 h). Bait stations were checked daily in the morning (0700–0900 h) and afternoon (1600–1800 h). Baits that had been consumed were recorded and replaced. In the afternoon of Day 2, all baits were replaced and re-located to the alternate side of the bait station. In total, each location had 45 baits of each type on offer (n = 5 waterbodies, n = 3 bait stations per site, n = 3 per bait station).

### Data processing and statistical analysis

#### Records of prey consumption

At each rebaiting event, we classified bait as eaten or uneaten and later identified the species of consumer by video. Our analysis in the current paper is restricted to cases where crocodiles were the consumers; other predators also took baits, notably raptors and varanid lizards (unpublished data). We created Generalised Linear Mixed Effects Models (GLMM) in SPSS Version 26 (IBM; Armonk, New York) incorporating ‘bait station’ nested within ‘waterbody’ as a random factor to account for pseudoreplication and repeated measures. We used Pearson’s correlation coefficient to test for significance (P < 0.05). To investigate any biases associated with the apparatus, we tested if the side of bait deployment affected the probability of bait consumption (eaten versus uneaten) using a GLMM with a binomial distribution and logit function. To investigate how crocodile foraging location (‘bank’, ‘water’s edge’, ‘directly over water’) was influenced by baiting period (day/night) and/or toad invasion history (toad presence versus absence at the site), we used a multinomial full factorial GLMM with a generalised logit function. Finally, to test if the probability of baits being eaten was influenced by toad invasion history (presence/absence of toads at the site) and/or bait type, we ran a full factorial GLMM with a binomial distribution and logit function.

#### Bait investigation on remote cameras

For all videos containing crocodiles in frame, we ran a chi-squared (contingency table) analysis to compare the proportion of baits that were investigated and subsequently consumed *versus* subsequently rejected for each bait type within our toad-sympatric population of crocodiles. Any direct interaction (i.e., sniffing, touching, or head purposefully moving within 20 cm of the bait) was classified as an ‘investigation’. If a crocodile did not ‘consume’ the bait after this interaction, the encounter was classified as a ‘rejection’. In addition to the total number of baits that were consumed by the end of the study, this measure provided visual evidence of any bait preferences or more importantly, active aversions.

#### Prey-handling behaviour of crocodiles

To understand the risks of consuming cane toads at different locations, and whether crocodiles could compensate for this in any way, we scored remote camera videos of bait consumption in the field to record: (i) bait type, (ii) where the crocodile seized the bait (bank, water’s edge, directly over water), (iii) where the crocodile eventually consumed the bait (land vs water), (iv) time elapsed (in seconds) between seizing a bait and consumption, and (v) time elapsed between siezing a bait and head submergence in water. In addition, once the bait had been seized, any events that included head movements by the predator that might facilitate ‘washing’ of prey and thus, dilution of cane toad toxin, were also recorded as ‘washed’ or ‘not washed’. Time data were log-transformed prior to statistical analysis to achieve normality and homogeneity of variance. We used ANOVAs, chi-squared tests, and regression analyses to interpret patterns. All non-GLMM analyses were done using JMP 14.2.0 (SAS Institute; Cary, North Carolina).

### Ethics statement

This study was approved by the Animal Ethics Committee of Macquarie University (2019/02-4) and conducted under to research permit number FO25000052-2 from the Department of Biodiversity, Conservation and Attractions (DBCA). Toad carcasses were sourced from the cane toad euthanasia program administered by the Western Australian state government. All protocols were governed by Australian Codes of Conduct for the Care and Use of Animals for Scientific Purposes. Access permission was granted by the DBCA, Indigenous Traditional Owners from the Bunuba people and Miriuwang Gajerrong people, and by private landowners. The authors complied with the ARRIVE guidelines.

## Results

### Summary of results from bait trials

Over the course of the field trials, we deployed 844 baits, and captured 1918 videos with crocodiles in frame (Fig. 1). We determined that there was no significant bias associated with the apparatus itself, as the side that bait was positioned did not influence whether or not the bait was eaten (F_1,1307_ = 0.25, P = 0.61).

### Bait type

**Figure 2:**
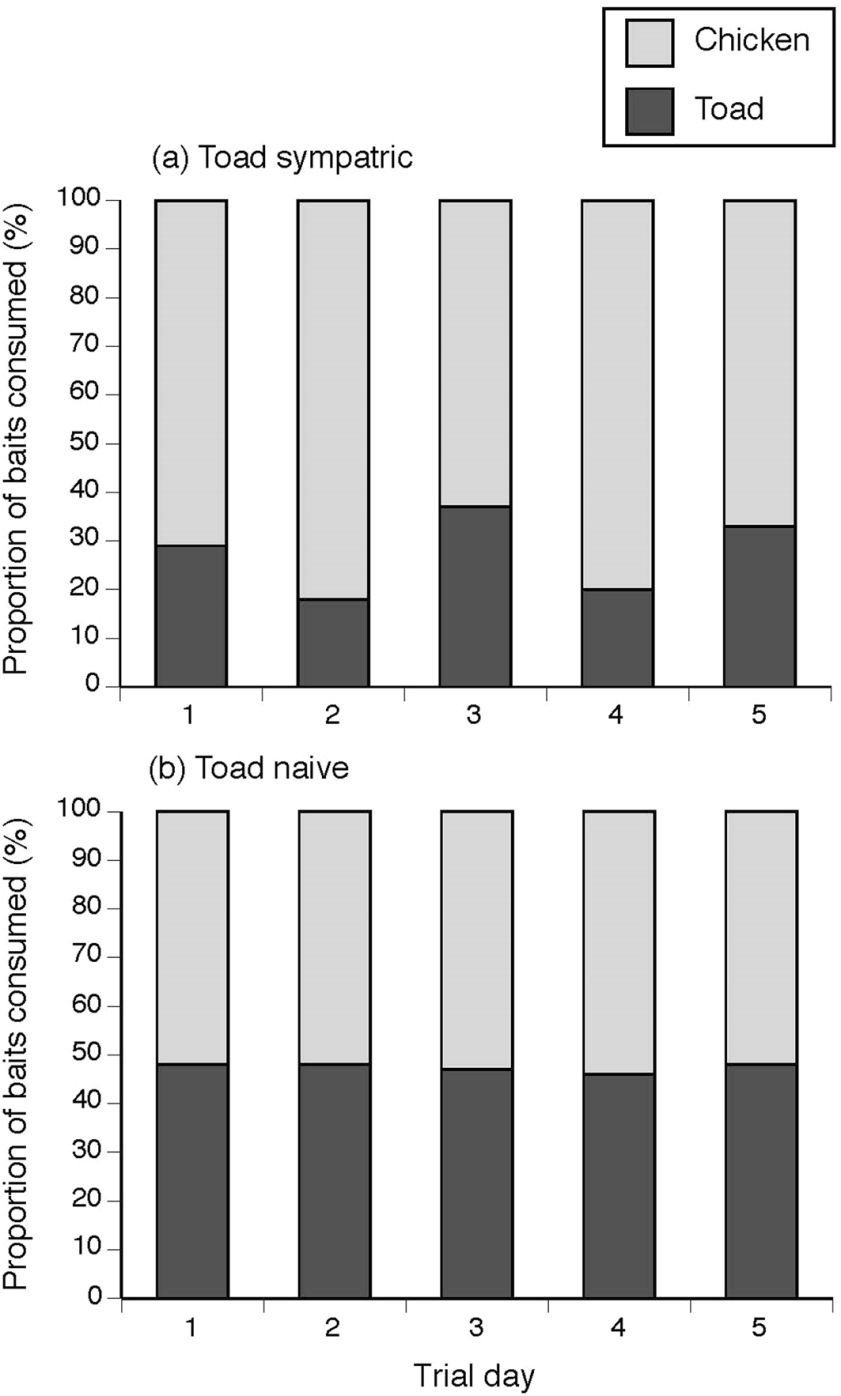
Proportion of chicken *versus* cane toad baits eaten by freshwater crocodiles (*Crocodylus johnstoni)* over the trial period by (**a**) crocodiles from toad-sympatric populations and (**b**) crocodiles from toad-naïve populations.

**Figure 3:**
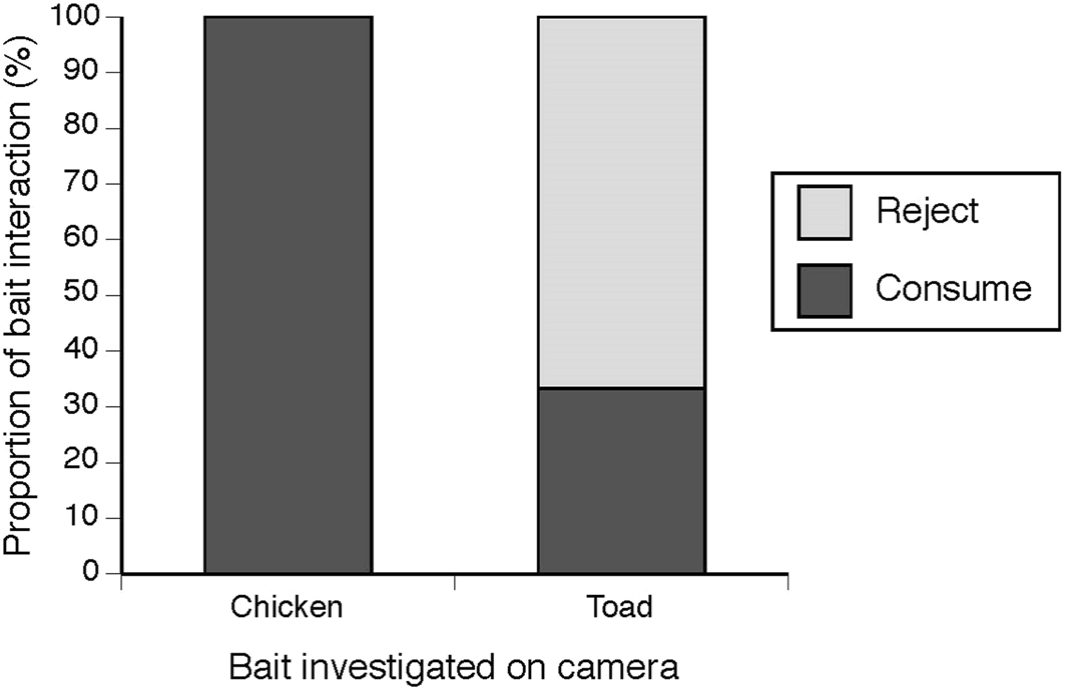
Analysis of reactions (consume or reject) of toad-sympatric populations of freshwater crocodiles (*Crocodylus johnstoni*) to chicken and cane toad baits, using data collected by remotely-triggered video cameras. Rates of consumption of toad *versus* chicken baits were equal in toad naïve populations (not shown).

Of all baits deployed across both trials, 49% of chicken baits and 40% of toad baits were consumed by crocodiles, but with a difference in bait uptake between sites where crocodiles were toad-sympatric *versus* toad-naïve. At the toad-absent sites, crocodiles took 290 chicken baits and 262 toad baits. At the toad-present site, 19 chicken baits and 5 toad baits were taken. Hence, the probability of baits being eaten was affected by the interaction between toad presence and bait type (toad vs chicken; F_1,1305_ = 4.76, P = 0.029). Toad-naïve crocodiles ate toad and chicken baits in equal numbers, but toad-sympatric crocodiles ate fewer toad baits than chicken baits (Fig. 2). Video analysis showed that this bias reflected active rejection of toad baits; that is, many of the crocodiles that investigated and/or seized toad baits did not consume them. In video recordings of toad-sympatric crocodiles investigating accessible baits, only 33% of toad baits were consumed (n = 2 of 6) compared to 100% of chicken baits (n = 7; X^2^ = 6.74, n = 13, P = 0.009; Fig. 3).

### Bait location

**Figure 4:**
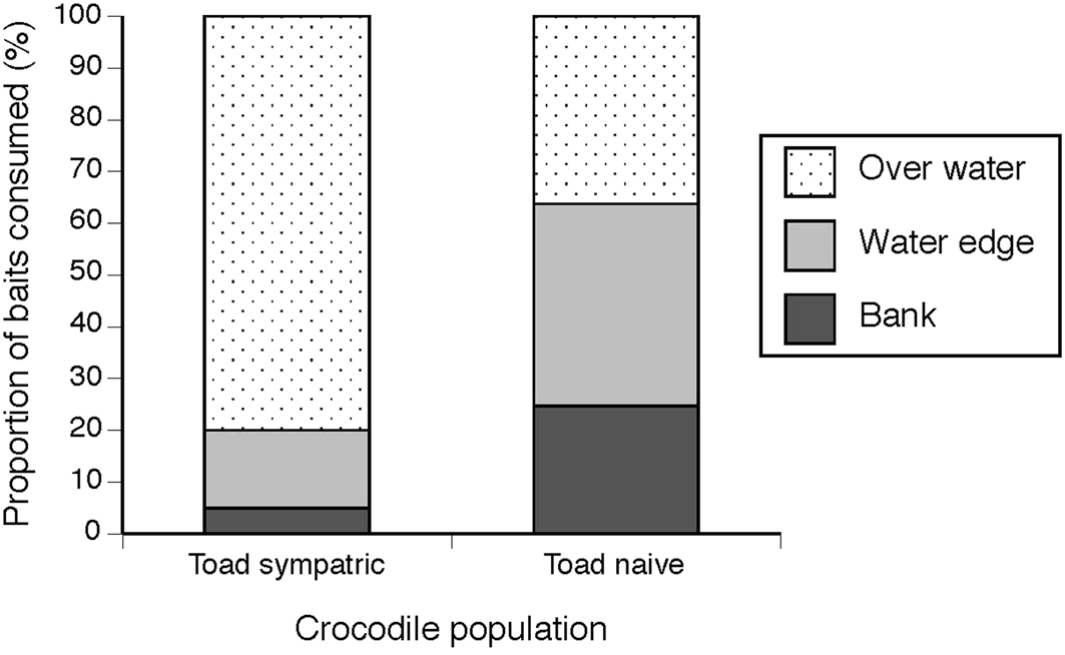
Location of baits consumed by freshwater crocodiles (*Crocodylus johnstoni*) from toad-sympatric and toad-naïve populations.

Crocodiles sometimes ate baits located directly over the water and sometimes ate baits located on land (water’s edge and on the bank) (See Fig. 1). The foraging locations where crocodiles consumed baits differed between field sites with *versus* without cane toads (F_2,581_ = 5.37, P = 0.005; Fig. 4). In toad-naïve populations, crocodiles took baits almost equally from all locations (over water n = 218, 39%, water’s edge n = 201, 36%, bank n = 138, 25%; Fig. 4). In contrast, crocodiles in toad-sympatric populations took baits primarily located over water (n = 20, 83%) in preference to baits located at the water’s edge (n = 3, 13%) or on the bank (n = 1, 4%). The numbers of baits taken from different locations did not differ significantly among baiting periods (day compared to night: F_2,581_ = 0.77, P = 0.47; interaction between baiting period and toad presence: F_2,581_ = 0.53, P = 0.59).

### Prey handling behaviour

We analysed 145 videos of crocodile prey handling behaviour from the toad-naïve population. Irrespective of where the bait was originally seized, crocodiles usually consumed baits in the water. The majority of crocodiles that took baits from the bank (76%) or from the edge of the water (97%) returned to the water to consume them. The time taken to swallow a bait did not differ significantly among baiting locations; most events were very quick (mean 15.0 s; F_2,121_ = 0.25, P = 0.78). Only two bait-taking events lasted > 1 min from the seizing of bait to swallowing. However, the average time between seizing a bait and submergence of the predator’s mouth (and thus the bait) in water was longer for crocodiles that took baits from the bank (mean: 7.4 s, range 2.79–32.9 s) than for those taking baits from the water’s edge (mean 2.2 s, range 0.4–19.2 s) or over the water (mean 2.0 s, range 1.0–5.0 s; F_2,50_ = 19.31, P < 0.001).

Bait type influenced whether or not a crocodile engaged in prey ‘washing’ behaviour (side-to-side movement of jaws; see Supplementary Video S1). Toad baits elicited significantly more ‘washing’ behaviour (49%) than chicken baits (28%) (F_2,144_ = 6.91, P = 0.03;

See S1 and S2 videos in Supplementary Materials). Crocodiles also ‘washed’ toad baits for longer than chicken baits (F_1,65_ = 4.37, P = 0.04).

## Discussion

Teasing apart the factors that influence prey choice and foraging tactics in the wild poses formidable logistical challenges because of multiple confounding features. For example, a particular type of prey may be rarely consumed not because of predator aversion, but because that prey type is more difficult to find or to capture than some other kind of prey^22^. Similarly, predators may key in on specific types of prey based on dietary preferences, prey size, or abundance^23–25^. The method of bait deployment that we adopted circumvents many of those problems, by standardising prey abundance, observability, and ease of capture by the predator. Under these conditions, free-ranging crocodiles from toad-sympatric *versus* toad-naïve populations showed substantial differences in foraging tactics and bait choice. In toad-naïve populations, crocodiles took equal numbers of treatment (toad) baits and control (chicken) baits, and frequently took baits located on land as well as over water. In contrast, crocodiles in toad-sympatric populations generally avoided toad baits in all locations and foraged primarily in the water rather than on land. Both of these shifts—in prey types and foraging locations—conceivably reduce the vulnerability of crocodiles to fatal ingestion of highly toxic cane toads.

The relatively rapid (< 8 years) development of aversion towards cane toads as prey, reflected both in the decreased proportion of toad baits consumed and by active rejections on camera, is unsurprising. Research on captive freshwater crocodiles reported rapid aversion learning in response to an initial non-fatal encounter with cane toads as prey^26^. Studies of other vulnerable predators (e.g., red-bellied blacksnakes, *Pseudechis porphyriacus*; common planigales, *Planigale maculata*; yellow-spotted monitors, *Varanus panoptes*) have shown that toad colonisation can induce a rapid and long-sustained aversion to their consumption as prey^1,27,28^. At least two mechanisms may underpin the elimination of cane toads from diets of these predators: behavioural plasticity (conditioned taste aversion) and natural selection (higher mortality of individuals with a genetically based propensity to consume toads^1,9^). Studies on newly hatched, and hence, toad-naïve, offspring of freshwater crocodiles from a range of sites (including locations where toad-induced mortality was high and others where it was not) did not reveal any geographic variation in their propensity to consume cane toads^26^. Thus, the ability to rapidly learn taste-aversion to cane toads may explain the shift in prey preferences of crocodiles following the arrival of toads.

**Figure 5:**
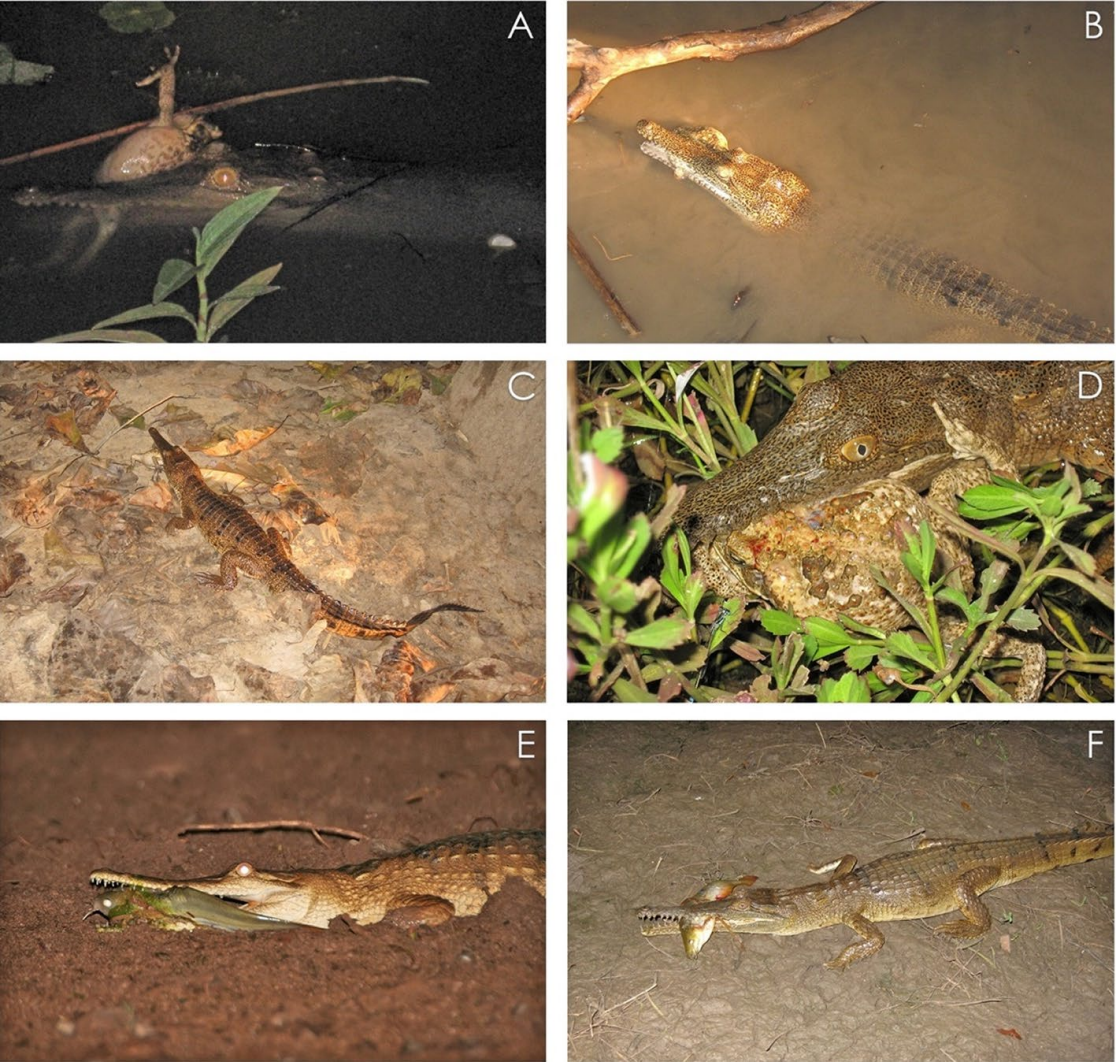
Freshwater crocodiles feeding on adult cane toads in the water (**A**,**B**) and on land (**C**,**D**). It is common for *C. johnstoni* crocodiles to consume prey on land, even if the prey is captured in the water (**E**: a catfish; **F**: a small barramundi). Photographs by R. Somaweera.

The divergence in foraging behaviour of crocodiles between our two regions (toad invaded *versus* uninvaded) is more novel and may support the hypothesis that crocodiles experience a higher risk of fatality when they forage for toads on land. Our video analysis of prey handling behaviour confirms that crocodiles do “wash” recently seized toads, more often and for longer than occurred with non-toxic (chicken) baits. Presumably then, crocodiles recognise the unpalatability of toads and attempt to eliminate potential toxicity by flushing their mouths with water. This option is not immediately accessible to a predator that seizes its prey on land, and even a few seconds’ delay in returning to the water might be enough to increase susceptibility to this fast-acting poison^8^. In our study, 25% of crocodiles that took baits on land did not return to the water to consume them; and this behaviour may be especially risky. Interestingly, consuming prey in the water is not the default foraging behaviour for larger crocodilian species such as *C. johnstoni*, that often consume prey on land^29^, even with aquatic items such as fish (Fig. 5). This comparison further supports the idea that naïve crocodiles perceive toads as unpalatable and return to the water to compensate for this.

Other species of predators also employ behavioural mechanisms akin to ‘washing’ when consuming toxic prey^16^. For example, the slender loris sneezes, slobbers and urinates on poisonous invertebrates prior to consumption^30^. Similarly, otters detect the toxins of novel prey *Bufo spinosus* and avoid dermal toxin glands by skinning and washing carcasses prior to consumption (despite never having encountered amphibians previously)^31^. Toxins are often bitter and unpalatable, facilitating detection by predators^16^.

The proximate mechanisms underlying the apparent shift to aquatic rather than terrestrial foraging remain unclear. As for dietary preference, a shift in foraging tactics might be either learned or genetically based. Tests on captive-raised crocodiles from different populations could address this question but would be challenging logistically. Crocodiles show strong ontogenetic shifts in their choice of foraging sites^19^ and thus, offspring from captive-raised clutches would need to be maintained for several years in captivity before their choice of foraging sites was assessed. However, other behavioural traits that correlate with terrestrial foraging might be easier to explore. We might expect that ‘bolder’ individuals would be more likely to leave the water to forage, if boldness is associated with increased exploration and willingness to forage in the open^32^. Previous studies have explored the link between predator boldness and cane-toad induced mortality. Not only do bolder varanid lizards forage in different locations^33^, they are more likely to eat novel prey types such as invasive cane toads and have poorer learning responses to taste aversion trials^10^. Although we did not test this aspect directly, behavioural traits of freshwater crocodiles could influence their (i) foraging locations, (ii) propensity to eat a cane toad and (iii) ability to learn from non-lethal interactions with cane toads. Standardised trials could assess behavioural syndromes relatively early in life^34^. In-situ behavioural assays of crocodiles across a larger spatial scale (in toad invaded *versus* uninvaded areas) may document wider intraspecific variation and clarify whether underlying ‘personality’ traits are indeed corelated with foraging behaviour. Such studies could investigate the propensity to forage terrestrially, and to return to the water with prey, both of which have implications for predator vulnerability. Interestingly, strong selection against specific foraging behaviours may have ecological ramifications, if shifts lead to higher rates of predation on aquatic and semi-aquatic species, and less on terrestrial species. When populations of apex predators change numerically or behaviourally, trophic cascades can ensue, with unpredictable impacts on meso-predators and prey species^35,36^.

As well as learned or genetically-based factors, environmental differences between study sites (such as bank steepness or availability of alternative prey) may deter crocodiles from climbing out of the water in search of prey. However, there was no overt variation in site topography among the waterbodies selected (data not shown). Furthermore, terrestrial prey was more abundant in the toad-invaded region than the uninvaded region (unpublished data), the opposite pattern to what we would expect if prey availability was driving crocodile foraging tactics.

Other potential influences on bait uptake could be differences in densities of crocodiles at toad-naïve versus toad-sympatric sites, and/or a disproportionate influence of a small number of individuals that consumed multiple baits. However, animals were given ample opportunities to access baits (multiple locations, multiple bait stations, bait replenishment twice a day), such that there were always many more baits available than there were crocodiles. Although we could not identify individual predators, our video analysis confirmed that crocodiles of a wide range of body sizes visited stations at each location. Generally, toad-naïve sites had higher densities of crocodiles, potentially increasing rates of bait consumption based on numbers, or via increased competitive foraging. Nonetheless, the rate of bait uptake was low in toad-sympatric populations even with high densities of crocodiles. These patterns may reflect toad impact in two ways: (a) densities of crocodiles have decreased in toad-sympatric sites due to toad-induced mortality of crocodiles; and (b) the crocodiles most likely to survive the toad invasion are shy, neophobic, water-foraging individuals. Such animals may be less likely to engage with the novel apparatus that we set up, thereby decreasing uptake of baits. Irrespective of overall uptake, however, we found robust evidence of toad aversion in the toad-sympatric populations: the relative offtake of chicken baits increased relative to offtake of toad baits. This comparison of responses to the two types of bait confirms the influence of toads in these areas.

Our spatial sampling for the present study was not ideal, for logistical reasons. Ideally, we would use a Before-After-Control-Impact design, sampling multiple sites before and after the arrival of cane toads. Future research could replicate our work, at that larger spatial and temporal scale. However, if the impact of toads on crocodile foraging behaviour does not manifest until a few years after invasion, considerable logistical challenges will need to be overcome. For the present, we can confidently conclude that freshwater crocodiles within a toad-sympatric region foraged on land less frequently than did conspecifics in a toad-naïve region.

In summary, our data on foraging responses of crocodiles to standard baits revealed both of the patterns that we predicted. Following invasion by toxic toads, crocodiles tended to eliminate toads from their diet, and foraged less often in a habitat (on land) where consuming a toad was most likely to be fatal for the predator. Although we cannot confidently infer either causation or mechanisms for that divergence, our results suggest that further work on this topic would be of value.

Our data highlights the potential for an invasive species to modify multiple behavioural attributes of vulnerable native taxa. In the case of freshwater crocodiles, toad invasion has potentially induced shifts in foraging locations as well as prey selection. Other behavioural traits that affect foraging tactics, such as overall boldness, or prey-handling, such as washing, may have also been modified. Research on other taxa affected by invasive cane toads have shown comparable changes in predator behaviour. For example, native fish that benefit from discriminating toxic cane toads from non-toxic frogs may have evolved enhanced learning ability^3^. Dasyurid marsupials have switched their use of sensory modalities for stimulating prey-attack from purely visually based cues to chemical and visual signals^27^. Clarke et al.^37^ suggested that other aspects of encounters in the wild between toads and crocodiles (i.e., the part of the toad’s body that is grasped first) may affect opportunities for natural aversion learning and hence, modify demographic impact. Our research supports this inference. More generally, the arrival of a toxic alien taxa can impose strong pressure on native wildlife to adapt in ways that reduce rates of encounter with the invader, rates of consumption of the invader, or cause more subtle shifts in the contexts in which such encounters occur.

## Supplementary Information

Supplementary Video 1.

Supplementary Video 2.

Supplementary Figure.

Supplementary Information.

## Data Availability

The datasets relevant to the current study are available through the Dryad data repository: 10.5061/dryad.rv15dv490.
