# Supplementary figures and images for: Shifts in the foraging tactics of crocodiles following invasion by toxic prey
Source: Sci Rep. 2022 Jan 24;12:1267. doi: 10.1038/s41598-021-03629-6 (PMC8786828; doi:10.1038/s41598-021-03629-6)

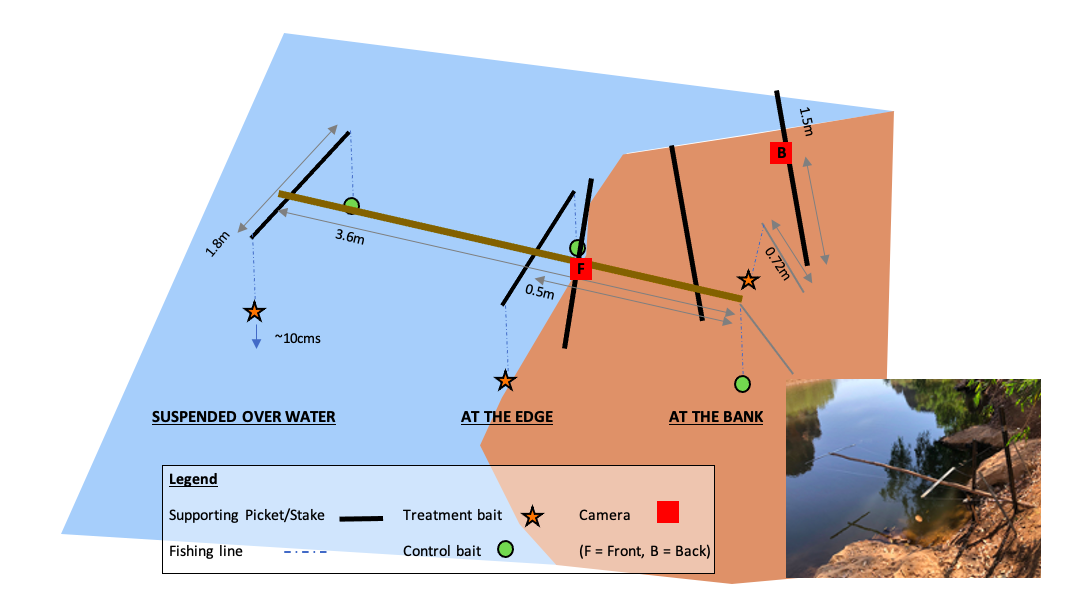

Supplement: Supplementary file 3 — Supplementary Figure. [file 41598_2021_3629_MOESM3_ESM.png]
