# Supplementary material for: Shifts in the foraging tactics of crocodiles following invasion by toxic prey
Source: Sci Rep. 2022 Jan 24;12:1267. doi: 10.1038/s41598-021-03629-6 (PMC8786828; doi:10.1038/s41598-021-03629-6)
Supplement: Supplementary file 4 — Supplementary Information. [file 41598_2021_3629_MOESM4_ESM.docx]

S1 – Supplementary Video 1

Freshwater crocodile climbing onto land to grab hanging chicken bait from baiting apparatus. Some sniffing of bait before active removal; crocodile then retreats back to the water to consume. Footage from remote camera traps at Windjana Gorge study site (2020).

S2 – Supplementary Video 2

Freshwater crocodile consuming toad bait (LHS) in the water after pulling bait off from baiting apparatus. Subtle cycling of water through mouth seen as an example strategy to assist in consuming/washing of toad baits. Footage from remote camera traps at Windjana Gorge study site (2020).
